# Multiple mechanisms for inbreeding avoidance used simultaneously in a wild ape

**DOI:** 10.1098/rspb.2023.1808

**Published:** 2023-10-18

**Authors:** Robin E. Morrison, Eric Ndayishimiye, Tara S. Stoinski, Winnie Eckardt

**Affiliations:** ^1^ Dian Fossey Gorilla Fund, Musanze, Rwanda; ^2^ Human Evolutionary Ecology Group, Department of Evolutionary Anthropology, University of Zurich, Winterthurerstrasse 190, 8057 Zurich, Switzerland

**Keywords:** gorilla, mate choice, kin recognition, dispersal, copulation patterns, paternity

## Abstract

Mating with close kin can have considerable negative fitness consequences, which are expected to result in selective pressure for inbreeding avoidance mechanisms, such as dispersal, mate choice and post-copulatory biases. Captive studies have suggested that inbreeding avoidance through mate choice is far less widespread than expected and may be absent where other mechanisms already limit inbreeding. However, few studies have examined multiple mechanisms of inbreeding avoidance simultaneously, particularly in the wild. We use 13 years of detailed dispersal, copulation and paternity data from mountain gorillas to examine inbreeding avoidance. We find that partial dispersal of both sexes results in high kinship in multimale groups, but that copulations between close kin occur 40% less than expected. We find strong kin discrimination in mate choice, with significant avoidance of maternal kin but more limited avoidance of paternal kin. We find no evidence for post-copulatory inbreeding avoidance. Our analyses support familiarity-based mechanisms of kin identification and age-based avoidance that limits mating between fathers and daughters in their natal group. Our findings demonstrate that multiple complementary mechanisms for inbreeding avoidance can evolve in a single species and suggest that inbreeding avoidance through mate choice may enable more flexible dispersal systems to evolve.

## 1. Background

Strong and stable social relationships have been linked with a range of fitness benefits across social mammals [1–5], and relationships with kin can provide even greater benefits [6–9]. Remaining in the same social group throughout life and interacting regularly with close kin could therefore increase fitness. However, reproducing with kin can substantially reduce fitness via inbreeding depression [10–12], and a variety of strategies have evolved in group-living animals to avoid reproducing with kin [13–15].

The most widespread form of inbreeding avoidance is dispersal by one or both sexes [14]. This considerably limits opportunities for close kin to reproduce [14,16] but leads to a range of costs including reduced kin benefits [17–19]. Sex-biased dispersal is common across social mammals, where females predominantly remain in their natal groups and males disperse around sexual maturity [13–15]. However, if dispersal is incomplete, with only some individuals of either or both sex dispersing, further mechanisms for avoiding inbreeding may be required, particularly where the risks of inbreeding depression are high [20,21]. These further mechanisms for inbreeding avoidance can be either precopulatory, involving recognizing and avoiding mating with kin, i.e. mate choice [16,20], or post-copulatory, such as sperm ejection, sperm selection and biased fertility [22,23]. The use of multiple inbreeding avoidance mechanisms simultaneously may enable more successful inbreeding avoidance, or this redundancy could be costly, with selection favouring one particular mechanism [21].

Studies examining mate choice in captive settings have suggested that inbreeding avoidance through mate choice is rare [20,24,25], while support from wild studies is more mixed [21]. A lack of inbreeding avoidance through mate choice could be due to low inbreeding depression (i.e. low costs to inbreeding) or alternative mechanisms for inbreeding avoidance, such as dispersal or post-copulatory biases, reducing the need for mate choice [21]. Few studies have been able to examine multiple mechanisms simultaneously in wild populations to understand the extent to which such mechanisms are used in combination [20]. However, a recent study in wild baboons has demonstrated a clear bias against mating with kin, particularly maternal kin, even when dispersal already results in few opportunities for opposite sex kin to mate [16]. This suggests that further studies examining multiple mechanisms for inbreeding avoidance simultaneously in wild populations are crucial for understanding the prevalence and evolution of different mechanisms of inbreeding avoidance, and how and when these mechanisms work in combination.

Mountain gorillas (*Gorilla beringei beringei*) represent a valuable study system in which to examine mechanisms of inbreeding avoidance. Gorillas, along with most of their ape relatives, go against the predominant trend of male dispersal and female philopatry [13–15], with both sexes usually dispersing [26,27]. Females usually transfer directly between groups which have overlapping home ranges [28]. Females can transfer multiple times across their lives and these transfers usually occur during intergroup encounters [29,30]. By contrast, males become solitary or join bachelor groups following dispersal, before attempting to attract females and form a group [31,32]. Mountain gorillas differ from other gorilla sub-species, by having an unusually flexible dispersal pattern, where only around half of individuals of either sex disperse from their natal group [30,33]. This results in variable group compositions of single-male and multimale groups and a variable mating system, showing both polygyny and polygynandry [34]. This differs considerably from other gorilla sub-species where groups are predominantly single-male and have a polygynous mating system [35,36].

Polygynous single-male groups are believed to be ancestral in the gorilla genus, as gorillas are highly sexually dimorphic, and no evidence for adaptations to sperm competition or paternity discrimination have been found [37–39]. Flexible dispersal, with a variable mating system and both single-male and multimale groups is believed to be a derived state that evolved relatively recently in mountain gorillas' evolutionary history [34,39]. Despite their presumably independent evolution, this flexible dispersal in mountain gorillas may represent the closest parallel in the great apes to dispersal patterns common across human evolutionary history, where either males or females could disperse from their natal group [40,41]. In human hunter–gatherer societies, strong incest taboos and exogamy rules exist, limiting rates of inbreeding despite this flexible dispersal resulting in close kin of opposite sex commonly residing together [42–45]. Similar rules surrounding mate choice in mountain gorillas may be critical to limiting inbreeding given the similarity in their dispersal patterns.

In mountain gorillas, the majority of copulations are initiated by females (63%) and forced copulations are extremely rare [46,47]. Males regularly ignore female solicitations (35.7%), particularly if more than one female is soliciting or if the female is pregnant [46,47]. Around 60% of reproductive mountain gorilla groups are single-male, although this has varied considerably over time [31,34,48]. These groups contain one adult male and multiple adult females that have been recruited to the group, who mate polygynously [31,34,48]. The remaining reproductive groups contain multiple adult males and females and have a polygynandrous mating pattern, with most females mating with multiple males each year [34]. In these groups, males other than the founding male (who is often no longer alive) almost exclusively grew up within the group, and breeding females consist of both those that were recruited from other groups and those that grew up in the group. Given this, kinship within many of these multimale groups is expected to be very high, raising the question of whether and how gorillas in these groups avoid reproducing with close kin. In addition to its evolutionary value, this question also has important consequences for the conservation of endangered mountain gorillas, which suffer from extremely low genetic diversity and a level of inbreeding exceeding even the most inbred human populations [49,50]. To understand the extent to which potential problems due to low genetic diversity may be further exacerbated by the incomplete dispersal of either sex in mountain gorillas, we must examine how often sexually mature opposite-sex kin are residing in the same group, and how effectively they avoid reproducing with each other.

Kin identification, a requisite for inbreeding avoidance via mate choice, primarily occurs through familiarity, age proximity (where close-in-age group members are more likely to have the same father) and/or phenotypic matching in primates [51,52]. In mountain gorillas, maternal kin are likely to be identified with relative ease through familiarity, due to the close social relationships maintained between mother and offspring, and between maternal siblings, throughout immaturity and often beyond [53–55]. However, identifying paternal kin is likely to pose difficulties in multimale groups, as females frequently mate with multiple males around conception [34] and male dominance rank is a better predictor of adult male–immature relationships than is paternity [56]. Furthermore, male immigration into a group is extremely rare [46,57] so all males in a female's natal group represent potential paternal kin.

Cruder age-based mechanisms may therefore be used to identify paternal kin. Female mountain gorillas in their natal group have rarely been observed mating with males old enough to be their father, leading to the suggestion that mating may be avoided between females and any male that was an adult in their group while they were maturing [46]. This is further supported by genetic analyses demonstrating that in five cases where females reproduced in a group where their father was the dominant male, he was not the father of their offspring, and fathers were less than 9 years older than mothers [58]. However, post-copulatory mechanisms rather than mate choice cannot be ruled out here, as we do not know how frequently father–daughter copulations occurred. Alternatively, mating between those close in age might be avoided, as in baboons, where age proximity is used as a cue for paternal siblings [59].

Phenotype matching, where individuals learn their own phenotypic attributes, such as odour or appearance, and compare these with potential kin, could provide a more accurate method for identifying paternal kin in multimale groups [51]. The capacity for phenotypic matching has been shown in other apes including chimpanzees which can distinguish facial similarities between kin [51,60] and humans in which kin recognition through both olfaction and visual similarity have been found [61–63]. Humans have also demonstrated the capacity to visually recognize kin in chimpanzees, western lowland gorillas and mandrills, demonstrating that facial resemblance is a reliable indicator of kinship in these species [64]. Given mountain gorillas' close evolutionary relationship to western lowland gorillas, and to a lesser extent chimpanzees and humans, viable indicators for phenotypic matching are highly likely to be present, e.g. through facial resemblance, although the extent to which these are used for kin recognition by gorillas themselves is unknown.

Finally, mountain gorillas could also rely on post-copulatory mechanisms for inbreeding avoidance which could result in few offspring being born as a result of inbreeding even if mating between relatives remains common. However, despite the breadth of studies on inbreeding avoidance through mate choice in primates, far less is known about post-copulatory inbreeding avoidance, which has more commonly been studied in birds, fish and arthropods [22,65–67].

In this study, we examine the extent to which inbreeding is avoided in multimale mountain gorilla groups through dispersal, mate choice and post-copulatory mechanisms. We use over five decades of data to examine dispersal patterns in each sex, detailing the process by which high proportions of opposite sex kin end up co-residing. We then use 13 years of detailed data on copulation patterns and offspring paternity in multimale groups to examine kin biases in copulation and reproduction, and the mechanisms by which kin are recognized.

## 2. Methods

### (a) Study population

Mountain gorillas in the Volcanoes National Park, Rwanda, have been monitored by the Karisoke Research Center of the Dian Fossey Gorilla Fund (Fossey Fund) since 1967. These gorillas are individually identifiable based on physical characteristics and habituated to human presence. Field staff have recorded demographic data, including group compositions, births, deaths and dispersals since 1967. This long-term monitoring has enabled the birthdates of 86.0% of gorillas born within this study population to be known to within a week, 9.9% to be known within a month, 3.5% to be known within three months and 0.6% to be known within six months (*n* = 344). Deaths are usually directly confirmed through observation of the corpse, with searches carried out for corpses of individuals that disappear from groups. Cooperation between organizations monitoring gorillas in Rwanda enabled all dispersals between gorilla groups within Rwanda to be confirmed. Where possible, dispersals into neighbouring countries were confirmed using genetic samples from census work [68,69]. Cases in which dispersal and death could not be distinguished were excluded from analyses (*n* = 3), except when the individual was severely ill prior to their disappearance in which case death was assumed (*n* = 2).

### (b) Natal dispersal

To examine dispersal from natal groups, we extracted the age at first dispersal for all individuals born within the study population that reached 6 years of age (*n* = 164, 80 females, 84 males). This age cut-off coincides with subadulthood and dispersal does not usually occur under this age, except when a group disintegrates [70]. In cases of group disintegration, immature individuals remained with a large proportion of their natal group and these were therefore not considered as cases of dispersal. We ran a Cox proportional hazards model predicting natal dispersal across age by sex, and fitted Kaplan–Meier curves using the ‘Survival' R package version 3.2-13 [71]. Of the 164 gorillas examined, 89 dispersed from their natal group and the remaining 75 were right-censored in the analysis, as they died before dispersing from their natal group or were still alive in their natal group at the end of the study (2021).

Mean female age at first birth and mean male age at reaching dominance were calculated to examine how the timings of these events mapped onto dispersal. Females reproduce consistently across their adult lives [72], while male reproduction varies across the lifespan [58,73]. Mean male age at reaching dominance was therefore examined as an indicator of when males are likely to reach their maximal rate of reproduction [58,73]. Mean female age at first birth was estimated by extracting age at first birth for all females that had been observed from less than 8 years old until their first birth (*n* = 59). Mean male age at first dominance was estimated by extracting the age of each male when they first (a) became the only adult male of their group or (b) became the highest ranked adult male in their group, for all males observed from less than 12 years until becoming a dominant male (*n* = 29). Dominance ranks in multimale groups from 2003 onwards were calculated using the Elo rating method [74] based on displacement and avoidance in the R package ‘EloRating' version 0.43 [75,76]. Prior to 2003, dominant males in multimale groups were identified solely through researchers' observations (data required to calculate Elo ratings were not consistently recorded). However, there is strong agreement between current Elo ratings and researcher observation.

### (c) Copulation data

Between 2003 and 2015, the Fossey Fund monitored between 3 and 12 gorilla groups for up to 4 h each day. During monitoring, copulations were recorded both ad libitum (i.e. whenever observed) and as part of the focal follow protocol. All recorded copulations between 2003 and 2015 from the ad libitum and focal data were combined to produce a single mating dataset including all observed copulations. Data were then extracted from all multimale groups (*n* = 9), defined as those with more than one sexually active adult male (greater than 9 years in age) [58]. Focal data are expected to provide a more accurate measure of the frequency of an event than ad libitum data, as a single individual is closely followed for a period of 50 min, and detailed behavioural data recorded [77]. Ad libitum data collection involves recording key behaviours for all group members simultaneously. Although this method has lower accuracy for estimating the frequency of events, it enables a far greater volume of data to be collected. Furthermore, copulations are usually accompanied by specific vocalizations and are therefore less likely to be missed than other events [47]. By combining copulations from both focal (*n* = 1165) and ad libitum (*n* = 3334) datasets, we can better understand copulation patterns within gorilla groups. Given the frequency with which copulations were recorded for focal and non-focal individuals, we estimated that copulations were 7.14 times more likely to be observed for a given pair while one of them was the focal individual in a focal follow, compared to when neither was a focal individual but researchers were present in the group.

In each year, in each multimale group, we examined copulations that were observed between opposite-sex pairs in which the female was over the age of 6 years (*n* = 71) and the male was over the age of 9 years (*n* = 52) by the start of the year, and both individuals were present in the same group for the entire year. These ages are consistent with female menarche and the beginning of male sexual maturity [46]. We extracted the total number of copulations observed between the pair during the year. The number of hours of ad libitum and focal observation for each pair in each year was also calculated. Groups with fewer than 700 h of observation in a year were excluded. In the remaining sample, the mean (s.d.) number of ad libitum observation hours was 1327.4 (±235.4), and the mean (s.d.) number of focal observation hours was 60.0 (±38.7) per dyad annually.

Kin relationships between each dyad were inferred based on the mother and father of each individual. All individuals' mothers were known, as they had been observed since birth. Fathers were known for only 54.9% of females and 86.5% of males based on previous genetic analyses [58]. Each co-resident, opposite-sex dyad in each year was assigned to one of five kin categories: mother–son (*n* = 82), father–daughter (*n* = 93), maternal siblings (*n* = 60), paternal siblings (*n* = 301), full siblings (*n* = 27), none (*n* = 685) and unknown (*n* = 1101). ‘None' included only pairs where the male could be ruled out as a father, son or sibling. ‘Unknown' included females whose paternity was unknown and therefore paternal kinship (either father or sibling) could not be ruled out. Excluding dyads of unknown kinship left a remaining sample size of 1248 dyads.

### (d) Reproduction data

All offspring of known paternity born within the study population, with estimated dates of conception (255 days prior to birth) between 2003 and 2015, were extracted from the demographic data (*n* = 44). Every known parent pair had been observed copulating at least once in the six months either side of conception and 77% had been observed copulating in the one month either side of conception, indicating that copulation data provided good coverage of actual mating events. Kinship of parent pairs was categorized into mother–son (*n* = 0), father–daughter (*n* = 0), maternal siblings (*n* = 2), paternal siblings (*n* = 5), full siblings (*n* = 0), none (*n* = 20) and unknown (*n* = 17). Excluding offspring whose parents' kinship was unknown left a remaining sample size of 27.

### (e) Kin discrimination in mate choice and reproduction

Previous research has demonstrated that females with dependent offspring (less than 3 years old) rarely copulate [34]. Females were therefore excluded from all analyses in years in which they had offspring with a mean age less than 3 years. To examine the extent of kin discrimination in mate choice, we used a negative binomial generalized linear mixed model (NB-GLMM), predicting the frequency with which co-resident opposite-sex pairs copulated during each year. Pairs with unknown kinship were excluded, leaving a sample size of 756 annual dyads. The model included kinship as a predictor, male ID and female ID as random effects, and a logged offset term for observation effort. Observation effort was calculated as the total number of hours during which *ad libitum* data were collected on the dyad plus 7.14 times the number of hours of focal monitoring for either member of the dyad. This accounted for the difference in rate at which copulations were observed between the data collection methods.

To examine the extent of post-copulatory kin discrimination in reproduction we compared the number of offspring born to dyads in each kinship category with the number of observed copulations by the mother, with males in each of the kin categories. All the mother's copulations in the one-month either side, and the six-months either side of the estimated conception date were extracted. The one-month time window was used to examine mating patterns when the female was expected to be most fertile, while the six-month time window provided a greater sample size with which to assess the female's general mating patterns. The distributions of (a) copulations within one month of conception (*n* = 77) and (b) copulations within six months of conception (*n* = 414) across each of the kin categories were compared with the distribution of parent pairs across kinship categories using Fisher's exact tests.

### (f) Mechanisms of kin identification for mate choice

We tested for the presence of the three most likely mechanisms of kin identification in mountain gorillas: familiarity, age and phenotypic matching. We defined two levels of familiarity: natal group (whether either individual was present in the natal group of the other when they were an infant) and maternal lineage (whether they were mother–son or maternal siblings). To examine age-based rules for avoiding kin, the age of each individual at the midpoint of each year was extracted. Male and female ages were then *z*-scored prior to analysis. To examine phenotypic matching, we hypothesized that overall, phenotypic similarities should be correlated with genetic relatedness, regardless of whether genes were maternally or paternally inherited [78]. We therefore used estimated relatedness: 0.5 for mother–son, father–daughter and full sibling pairs, 0.25 for paternal and maternal siblings and 0 for non-kin.

We predicted the frequency with which co-resident opposite-sex pairs copulated during each year (*n* = 756) using an NB-GLMM. The model included natal group, maternal lineage, male age, female age, the interaction between male and female age, and estimated relatedness as predictors. Male ID and female ID were included as random effects, and observation effort was included as a logged offset term, as described above. Females with dependent offspring and pairs of unknown kinship were excluded. The same model, but without estimated relatedness as a predictor, was also run on the larger dataset that included dyads of unknown kinship (*n* = 1399).

We then ran separate models for females in their natal group and those that had dispersed to examine whether they were using different mechanisms. Due to the smaller sample sizes, models with estimated relatedness as a predictor including only dyads of known kinship could not converge. We therefore used the larger dataset including dyads of unknown kinship and did not examine estimated relatedness. We ran NB-GLMMs for natal (*n* = 718) and dispersed (*n* = 681) females, predicting the frequency with which co-resident opposite-sex pairs copulated during each year. Each model included maternal lineage, male age, female age and the age interaction as predictors. Natal group was included as a predictor in the model for dispersed females but not for natal females as all natal female dyads had been present together in the female's natal group when she was an infant. Both models included male ID and female ID as random effects and an offset term for observation effort. The natal female model was also run excluding any females under the mean age of first birth (10.11 years) to verify that effects were not being driven solely by young infertile females.

We ran all NB-GLMMs in the ‘lme4' R package version 1.1-29 [79] and checked them against null models containing only random effects using AIC. All full models provided a significantly better fit than the null models when tested through ANOVA. Negative binomial models were used due to the large number of zeros in the data, as they include an extra parameter to directly model overdispersion.

## 3. Results

### (a) Dispersal and kin composition in groups

**Figure 1.**
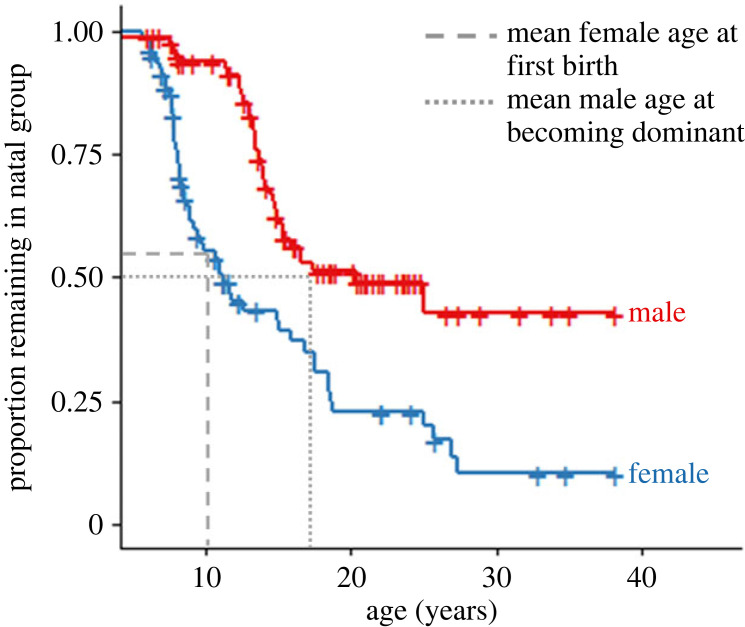
Kaplan–Meier curves depicting the proportion of males and females remaining in their natal group. *N* = 89 natal dispersals from 164 gorillas (80 female, 84 male). Crosses indicate individuals that died or are still monitored, and are yet to disperse (i.e. right-censored individuals). Dashed lines indicate the proportion of females in their natal group at the mean female age at first birth (10.11 years) and the proportion of males in their natal group at the mean male age at becoming dominant (17.13 years).

**Figure 2.**
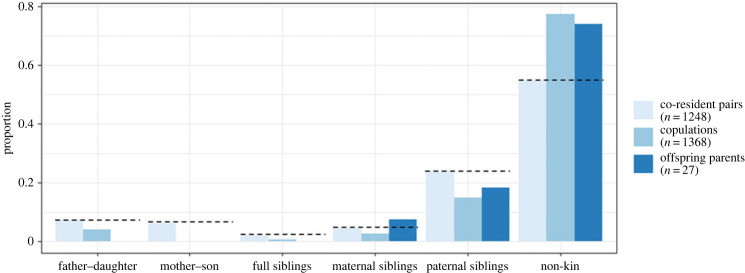
Opportunities to copulate (co-resident pairs), observed copulations and resulting offspring across kin categories. Opportunities to copulate represent opposite-sex pairs that resided in the same group over the course of a year. Observed copulations represent all copulations observed between these pairs. Offspring parents include all parents of offspring of known paternity conceived within the study period. Dashed lines indicate the proportion of copulations and offspring expected between pairs of each kin type if no kin discrimination were occurring. See electronic supplementary material, figure S1, for co-resident pair and copulation proportions broken down by group.

To quantify the extent of dispersal by either sex, we examined the proportion of males and females that resided in their natal group (i.e. had not dispersed) across age (figure 1). Female mountain gorillas were more likely to disperse and dispersed younger than males, resulting in a slight female bias to dispersal patterns (figure 1; Cox proportion hazards model, male dispersal: *z* = −4.463, *p* < 0.001). Mean female age at first birth was 10.11 years, at which point 47% had dispersed. At the mean age of first becoming a dominant male (17.13 years), a proxy for when males reach their maximal rate of reproduction, 50% of males had dispersed (figure 1). These dispersal patterns resulted in high kinship between mature co-resident, opposite-sex pairs of gorillas in multimale groups. Between 2003 and 2015, 41.7% (479 out of 1149) of pairs with known maternity and paternity had a close kin relationship (mother–son, father–daughter, full sibling or half-sibling; figure 2). This likely represents a slight overestimate of kinship within multimale groups, as females that transferred into the group, who are less likely to be close relatives, are overrepresented in the sample for which maternity and paternity is not known. Overall, known close kin represented 27.3% of pairs, those with no close kin relationship represented 28.5% of pairs, and those of unknown kinship represented 44.1% of pairs (*n* = 2349).

### (b) Kin discrimination in mate choice and reproduction

Using only pairs of known kinship (*n* = 1248), we examined patterns of kin bias in mate choice and reproduction. Although non-kin pairs represented 54.1% of the sample, 77.3% of copulations occurred between them and they produced 74.1% of offspring (figure 2). By contrast, 45.9% of pairs were close kin, but only 22.7% of copulations occurred between them, the majority of which were between paternal half-siblings (15.8%), and they produced only 25.9% of offspring (figure 2).

To examine the differences between opportunity to copulate and observed copulations statistically, we predicted the number of times each pair was observed copulating in a year based on their kinship (table 1). Mother–son copulations were avoided most strongly, with significant discrimination against copulating seen for all maternal kin categories. The number of observed copulations between father–daughter and paternal sibling pairs were not significantly lower than non-kin, indicating limitations in individuals' ability to discriminate paternal kin. However, although not statistically significant, copulations still occurred 45.1% less often in father–daughter pairs, and 37.6% less often in paternal siblings, than expected given patterns of co-residency. These reductions may still be highly relevant biologically in the reduction of inbreeding, despite the lack of statistical significance. Overall, this model showed strong support for mate choice-based mechanisms of inbreeding avoidance.

**Table 1.** Kin discrimination in copulation frequency. Negative binomial generalized linear mixed model (NB-GLMM) predicting the number of copulations observed between a co-resident mixed-sex pair of gorillas of known kinship, within a year (*n* = 756). The effect of each kinship category is shown relative to pairs with no close kin relationship.

	est.	s.e.	*Z* value	Pr(>|*z*|)
(intercept)	−7.573	0.222	−34.122	<0.001
father–daughter (*r* ≈ 0.5)	−0.394	0.366	−1.077	0.281
mother–son (*r* ≈ 0.5)	−3.388	0.803	−4.220	<0.001
full siblings (*r* ≈ 0.5)	−1.236	0.521	−2.373	0.018
maternal siblings (*r* ≈ 0.25)	−0.832	0.373	−2.228	0.026
paternal siblings (*r* ≈ 0.25)	−0.319	0.242	−1.318	0.188

To examine the differences between observed copulations and resulting offspring statistically, we compared the kinship of parents with the kinship between each offspring's mother and her mating partners within one month and within six months of conception (electronic supplementary material, table S1). The distribution of kinship categories across parent pairs did not differ from the mother's mating partners in the month either side of conception (Fisher's exact test: *p* = 0.076) or the six months either side of conception (Fisher's exact test: *p* = 0.144). If post-copulatory inbreeding avoidance was occurring, we would expect to find significantly fewer offspring with related parents than found in the mating history. By contrast, the proportion of offspring with maternal and paternal sibling parents were actually marginally (but non-significantly) higher than expected based on the mothers' mating patterns. This analysis did not support the presence of any post-copulatory mechanisms of inbreeding avoidance. We therefore focused our subsequent analyses on mechanisms of inbreeding avoidance through mate choice.

### (c) Mechanisms of kin identification for mate choice

We examined three mechanisms of kin identification for mate choice: familiarity, age and phenotypic matching, to predict observed copulation patterns. Familiarity was modelled with two variables: maternal lineage (mother–son and maternal sibling pairs) and natal group (whether either individual was present in the natal group of the other when they were an infant). Age was modelled by including both male and female age and the interaction between them. The potential for phenotypic matching to influence mate choice was modelled using estimated relatedness (i.e. 0.5 for mother–son, father–daughter and full sibling pairs, 0.25 for paternal and maternal siblings and 0 for non-kin). It is important to note the limitations of estimating relatedness in this way, as true values of relatedness for siblings are far more variable and this classification ignores any relatedness between more distantly related kin. The model showed strong support for familiarity and age-based mechanisms of inbreeding avoidance, but no support for phenotypic matching (table 2).

**Table 2.** Mechanisms of kin identification and inbreeding avoidance. NB-GLMM predicting the number of copulations observed between a co-resident mixed-sex pair of gorillas with known kinship, within a year (*n* = 756).

	est.	s.e.	*Z* value	Pr(>|*z*|)
(intercept)	−7.112	0.282	−25.291	<0.001
**familiarity**				
maternal lineage	−0.814	0.315	−2.583	0.010
natal group	−0.745	0.277	−2.686	0.007
**age**				
male age	0.508	0.153	3.311	0.001
female age	−0.302	0.108	−2.792	0.005
age interaction	0.383	0.110	3.487	<0.001
**phenotypic matching**				
estimated relatedness	−0.350	0.655	−0.534	0.594

**Figure 3.**
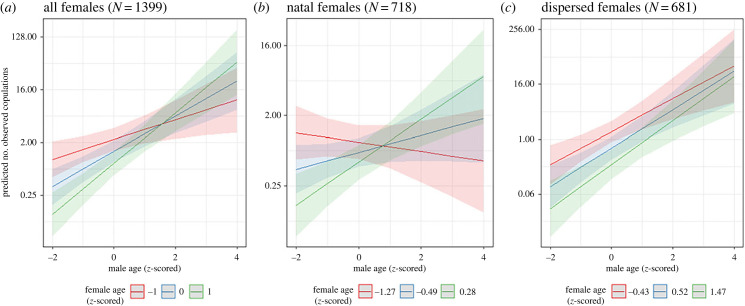
The interaction between male and female age on the number of times that a co-resident mixed-sex pair were observed copulating during a year for (*a*) all females, (*b*) natal females and (*c*) dispersed females. Female *z*-scored ages plotted represent the mean age in the sample (blue) and one standard deviation above and below (green and red). These correspond with (*a*) age 8.5, 17.4 and 26.3 years, (*b*) age 6.1,13.0 and 19.9 years and (*c*) age 13.6, 22.0 and 30.4 years.

Both maternal lineage and natal group were strong predictors of copulation frequency. Given that all pairs of the same maternal lineage were also present in the same natal group it suggests two familiarity-based rules are in play: limiting copulations with individuals from the natal group, and to an even greater extent, those from the same maternal lineage. Males were more likely to be observed copulating with increasing age, while females were less likely to be observed copulating with age (table 2). There was also a strong interaction between male and female age on copulation frequency. The strength of the increase in the mating frequency with older males was weaker, the younger the age of the female partner. As estimated relatedness did not predict copulation patterns when familiarity and age mechanisms were included, an additional model was run on the larger dataset including pairs of unknown kinship, predicting copulations from familiarity and age. This showed even stronger support for familiarity and age-based mechanisms of inbreeding avoidance (electronic supplementary material, table S2; figure 3*a*).

Given that males almost never transfer into a new group [46,57], females that remain in their natal group would have no mating partners if they avoided all males that were present during their infancy or were later born into the group. Furthermore, if the patterns of age-based avoidance shown in table 2 were a mechanism for avoiding mating between father–daughter pairs, this rule would only be beneficial to females in their natal group, as dispersed females are unlikely to reside in the same group as their father. We therefore ran two further models examining mechanisms of inbreeding avoidance separately in natal and dispersed females using the larger dataset including pairs of unknown kinship (table 3). Maternal lineage was a strong predictor of copulations by both natal and dispersed females who showed strong avoidance of copulating with maternal kin. Dispersed females did not avoid copulating with those in the natal group category. This is likely because these males were primarily those born into the group after the female arrived (i.e. she was present in their natal group when they were infants). They therefore were unlikely to represent kin unless they were her direct offspring.

**Table 3.** Mechanisms of kin identification and inbreeding avoidance in natal and dispersed females. NB-GLMM predicting copulations between co-resident mixed-sex pairs for (a) females in their natal group and (b) dispersed females. Natal group familiarity was not included as a predictor for natal females as all male partners had been present in the natal group. Sample included pairs of both known and unknown kinship.

	est.	s.e.	*Z* value	Pr(>|*z*|)
**(a) natal females (*n* = 718)**				
(intercept)	−8.195	0.237	−34.574	<0.001
**familiarity**				
maternal lineage	−0.661	0.283	−2.332	0.020
**age**				
male age	0.496	0.165	3.006	0.003
female age	−0.380	0.176	−2.161	0.031
age interaction	0.499	0.111	4.501	<0.001
**(b) dispersed females (*n* = 681)**				
(intercept)	−7.602	0.266	−28.539	<0.001
**familiarity**				
maternal lineage	−2.187	0.875	−2.498	0.013
natal group	0.197	0.334	0.591	0.554
**age**				
male age	0.899	0.208	4.313	<0.001
female age	−0.873	0.169	−5.180	<0.001
age interaction	0.145	0.119	1.224	0.221

Gorillas showed strong age-based mating preferences. Young, natal females, who could feasibly be the offspring of older male group members, were less likely to copulate with a male, the older that male was (figure 3*b*). This went against the trend seen in mid-age and older females who were more likely to copulate with a male the older they were. In dispersed females, the frequency of females of all ages copulating increased with the male partners' age, and younger females copulated less frequently overall (figure 3*c*). Females aged 6–10 years are often sexually active [34] but not yet reproductively active, as the mean age at first birth is 10.11 years. The natal female model was therefore rerun excluding females under 10.11 years, to verify that the interaction between male age and female age was not driven by dominant (and usually older) males avoiding mating with young infertile females [58]. This model (electronic supplementary material, table S3) was highly consistent with the previous model where younger females were not excluded, demonstrating that this pattern was not driven solely by young, potentially infertile females.

## 4. Discussion

We demonstrate that close to half of females (47%) disperse from their natal group prior to the mean age at first birth, and half of males (50%) disperse prior to reaching the mean age for first becoming dominant. These unusually flexible dispersal patterns result in high kinship in multimale mountain gorilla groups, where for those with known paternity, there was a more than 40% chance that an opposite-sex group member of reproductive age was a close relative. This partial dispersal may therefore further exacerbate the exceptionally low genetic diversity found in mountain gorillas' small and isolated populations [49]. These high kinship groups differ considerably to those found in most social mammals, where co-residency of opposite-sex close kin is largely prevented through death and dispersal [13–16]. However, they share similarities with human hunter–gatherer groups in which bisexual philopatry and dispersal result in opposite-sex kin frequently co-residing [80].

The high proportions of kin in multimale gorilla groups suggest there may have been strong selection for pre- or post-copulatory mechanisms of inbreeding avoidance. Our analyses found support for pre-copulatory inbreeding avoidance through mate choice but no evidence of post-copulatory inbreeding avoidance. We found no reduction in offspring resulting from inbreeding relative to that expected given females mating patterns in the period surrounding conception. These analyses were limited by the small number of offspring born into the population for which the paternity of the offspring and both parents were known. The potentially higher than expected number of offspring resulting from inbreeding between half-siblings (figure 2; electronic supplementary material, table S1) warrants further investigation when a larger sample size is available.

Mountain gorillas in multimale groups showed a significant bias against mating with all maternal kin (mother–son, full sibling and maternal sibling pairs). Although copulations between paternal kin were lower than expected given patterns of co-residency, occurring 45.1% less in father–daughter pairs, and 37.6% less in paternal siblings, than expected, our model detected no significant bias against copulating with paternal kin. This suggests that mechanisms of maternal kin identification in mountain gorillas have high accuracy, while mechanisms of paternal kin identification are more limited. As in baboons [16], mountain gorillas' limited ability to discriminate paternal kin may be a constraint on the avoidance of inbreeding.

We found strong support for familiarity- and age-based mechanisms of kin identification, but no support for mechanisms based on phenotypic matching used in inbreeding avoidance. Familiarity is likely to be a highly accurate mechanism for identifying maternal kin in mountain gorillas as offspring usually spend upwards of 6 years in close contact with their mothers and any close-in-age maternal siblings [53,55]. Familiarity is unlikely to be as reliable for identifying paternal kin, since females consistently mate with multiple partners [34], and rank rather than paternity predicts adult male–immature relationships [56]. It is unlikely that paternal kin could be discriminated from the wider group, although gorillas could still discriminate group members (potential paternal kin) from non-group members (unlikely to be paternal kin). Our model supports this, demonstrating that where available, mountain gorillas prefer copulating with individuals that were not present in their natal group when they were infants, similar to exogamy rules common in many hunter–gatherer societies [42–45].

Mountain gorillas also showed strong age-based preferences in their copulation patterns. The strong interaction between male and female age in copulations involving natal females, and the lack of such an interaction in dispersed females, provides good evidence that these age biases are a flexible behavioural strategy for inbreeding avoidance. Specifically, females in their natal group appear to be avoiding copulating with any male old enough to be their father, but females that have dispersed from their natal group are not applying this rule. In combination with preferences against copulating with individuals that were present in their natal group, these mechanisms, although far from perfect, likely contribute to the lower-than-expected frequencies of copulation between paternal kin, reducing rates of inbreeding in these high-kinship groups.

But why have more accurate mechanisms of paternal kin recognition, e.g. through phenotypic matching, not evolved in mountain gorillas? While more accurate estimates of genetic relatedness would be valuable for confirming this lack of phenotypic matching, one possibility is that this absence may be due to evolutionary lag, given the potentially quite recent evolution of multimale groups, and the occurrence of both single- and multimale groups [39,48]. However, inbreeding avoidance may also be constrained by a lack of unrelated alternative mating partners in some groups [25]. High male reproductive skew and long tenure length mean that close-in-age group members could all represent potential paternal siblings [58,73], while older males represent putative fathers [46]. Mating with a half-sibling may be one of the least unfavourable options for a natal female. If the inbreeding depression resulting from breeding between half-siblings is less costly than that associated with dispersal [17–19], remaining in the natal group may still represent the most successful strategy. This may be more common in mountain gorillas compared to other social mammals, given evidence for the purging of severely deleterious mutations in their recent history [49].

Another key question is when did inbreeding avoidance through mate choice in mountain gorillas evolve? Was it before or after mountain gorillas' switch from universal to partial dispersal of both sexes? Evidence from baboons demonstrates that inbreeding avoidance mechanisms can be present even when related individuals rarely have the opportunity to mate [16], suggesting that mate choice could predate changes in dispersal pattern. Kin recognition is common across primates, enabling a variety of benefits through kin-biased behaviours [81] and appears to be widespread across the gorilla genus [55,82]. In western lowland gorillas, which produce almost exclusively in single-male groups [32], familiarity-based kin recognition is likely to be highly accurate for both maternal and paternal kin. Genetic evidence suggests this kin recognition may be used to inform dispersal decisions [82] (but see [35]). The capacity to recognize kin through familiarity therefore likely predates mountain gorillas' flexible dispersal patterns and the resulting multimale groups. However, age-based mating biases are unlikely to be beneficial in other gorilla sub-species, given that single-male groups and complete dispersal are the norm [29,32]. In western lowland gorillas, all females were found to disperse before sexual maturity even if the adult male of their group was not their father [29]. This suggests these age-based mating biases may have evolved with or directly following the shift from universal to partial dispersal in mountain gorillas.

Overall, we show that familiarity and age-based mechanisms of kin identification limit inbreeding in mountain gorillas, despite partial dispersal resulting in opposite-sex kin frequently co-residing. However, mating between paternal kin is still common, and mountain gorillas' switch to partial dispersal by both sexes may have contributed to the substantial decline in their genetic diversity over the last 100 000 years [49]. Further research on the fitness consequences of inbreeding in current populations would be valuable for ongoing mountain gorilla conservation. We demonstrate how multiple inbreeding avoidance mechanisms: partial dispersal and familiarity- and age-based rules of mate choice, can be used flexibly and simultaneously in a wild population. Rather than the use of multiple mechanisms resulting in redundancy [21], inbreeding avoidance through mate choice may remove the necessity for one or both sexes to obligately disperse. Mate choice could enable individuals to access benefits associated with remaining in the natal group [8], while limiting the costs of inbreeding. Alternatively, where the benefits of remaining in the natal group are low, individuals may still choose to disperse, avoiding the reduced but still significant risk of inbreeding. The capacity for inbreeding avoidance through mate choice may therefore represent an important step in the evolution of more flexible dispersal strategies, such as those seen in humans and mountain gorillas.

## Data Availability

Data underlying these analyses can be found at https://doi.org/10.5061/dryad.zcrjdfnk3 [83]. Supplementary material is available online [84].
